# Assessment of the knowledge and attitude of infants’ mothers from Bushehr (Iran) on food security using anthropometric indicators in 2016: a cross-sectional study

**DOI:** 10.1186/s12889-018-5531-5

**Published:** 2018-05-11

**Authors:** Sedigheh Yeganeh, Niloofar Motamed, Saeid NajafpourBoushehri, Maryam Ravanipour

**Affiliations:** 1 Department of Nursing, School of Nursing and Midwifery, Bushehr University of Medical Sciences, Rishehr Street, PO Box 7518759577, Bushehr, Iran; 2 Department of Community Medicine, School of Medicine, Bushehr University of Medical Sciences, Moallem Street, PO Box 7514763448, Bushehr, Iran; 3 Department of Nutrition, School of Health and Nutrition, Bushehr University of Medical Sciences, Rishehr Street, PO Box 7518759577, Bushehr, Iran; 4 The Persian Gulf Tropical Medicine Research Centre, The Persian Gulf Biomedical Sciences Research Institute, Bushehr University of Medical Sciences, Bushehr, Iran; 5 Department of Nursing, School of Nursing and Midwifery, Bushehr University of Medical Sciences, Rishehr Street, PO Box 7518759577, Bushehr, Iran

**Keywords:** Anthropometry, Complementary feeding, Food security, Obesity, Stunting, Iran

## Abstract

**Background:**

Among the main elements of food security, in terms of food usage, are knowledge and attitude. These are particularly important during the initial two years of a child’s life. The present study was conducted in 2016 and aimed to assess the knowledge and attitude of infants’ mothers from Bushehr (Iran) towards food security using anthropometric indicators.

**Methods:**

The present cross-sectional, descriptive-analytical study was performed on 400 mothers of children aged 1-2 years in Bushehr, Iran. Data were collected using a 20-item knowledge questionnaire (CVR = 0.95, CVI = 0.95, and reliability 0.7), a 26-item attitude questionnaire (CVI = 0.94, CVR = 0.91, and reliability 0.76), and a 16-item Radimer/Cornell questionnaire, which were completed by all mothers. Anthropometric indicators of children, including height-for-age, weight-for-age, and weight-for-height were also measured in accordance with the z-score benchmark of the World Health Organization.

**Results:**

A positive and significant relationship was found between knowledge and attitude (*r* = 0.26, *P* = 0.0001) as well as between knowledge and household food security (*r* = 0.11, *P* = 0.02) in complementary feeding. Approximately 26% of the studied children fell under the risk category of overweight to obese. A significant relationship was found between inadequate knowledge of the mothers and height-for-age (OR = 4.87, *P* = 0.001) and weight-for-height (OR = 2.33, *P* = 0.04) indices, as well as between the negative attitude of the mothers and weight-for-height index (OR = 2.91, *P* = 0.03).

**Conclusions:**

The knowledge of food security purely relates to the dimension of the household food security of a family and not to the individual/child level of food security. It seems that the knowledge of a mother, as a positive factor, does not support child’s food security when the severity of household insecurity triggers the child’s hunger and food inaccessibility. Also, inappropriate knowledge and negative attitude towards food security were associated with an increased risk of obesity. Increased weight, in addition to being affected by the knowledge and attitude of the mothers, is probably also influenced by the incorrect conduct of the mothers. Further investigation on this topic is recommended.

**Table:** 

✓ The prevalence of approximately 25% of overweight and obesity in 1-2 years old infants from Bushehr. ✓ Increased risks of stunting and obesity in infants due to inadequate knowledge of food security by the mothers. ✓ Increased risks of obesity in infants due to the negative attitude towards food security by the mothers. ✓ Application of the role of household food insecurity knowledge to correct food insecurity and prevent child nutritional harm.	

## Background

The effect of nutrition and food security on human health, particularly during the early years of childhood, is evident [1]. The initial 2 years of life are the most important contributor to the growth and development of a child. Food insecurity during this period is the source of many chronic diseases during adulthood [2, 3]. In 2015, one out of every 6 American children experienced food insecurity [4]. Food insecurity among the Iranian children is reported as high as 67% [5].

Feeding during the initial 2 years of life is termed complementary feeding [6] and is associated with many factors such as the knowledge and attitude of a mother [7]. Mothers play an important role since they are generally responsible for providing and allocating food to the family [8]. Aside from financial capacity, as one of the components of food security, studies have shown that children are better fed if their mothers have a sufficient knowledge of feeding methods [9]. Hence, an appropriate attitude leads to a coherent feeding habit and behavior [10]. A study by Gholizadeh et al. (2017) on rural households in Kermanshah (Iran) indicated feeding knowledge as one of the main causes affecting food insecurity. They suggested that improving the feeding knowledge of households is a way to improve household food security [11]. On the other hand, at the household level, food security has different components and the amount of food being purchased and consumed is a major element [12]. One of the main components of providing food security - in terms of utilization - is knowledge, culture, and literacy. It allows the family members to easily base the choice of food on what they like (desirability), what they can afford (affordability), and what they need (adequacy) [8, 13, 14]. It is noteworthy to mention that a study in three southern provinces of Iran, including Bushehr, indicated the lack of knowledge as a major issue in improving nutrition [15].

Food insecurity (i.e. inadequate feeding in complementary feeding [16]), is one of the most undesirable forms of child neglect and, if prolonged, will have a direct effect on the weight and height of a child [1]. Indirect means (e.g. determining the extent of food insecurity, recognizing deficiencies, and providing appropriate nutrition status assessment) are the preferred and most desirable methods to understand how to feed children [17, 18]. There are various methods to examine feeding status (e.g. clinical, biochemical, dietary, etc.) [19] among which anthropometry is the preferred method [20]. Anthropometric method is the determination of an individual’s physical size and its relation to the standards, which indicates growth and development [21]. By recording the size of a child, one can determine his/her progress and feeding, individually monitor the health and feeding status of the child, and determine the feeding status of the population [22]. A nationwide survey on the development of children in Iran (1998) showed that a large percentage of children in the country suffer from growth decline. It began from the age of 6 months (due to inappropriate feeding pattern and habits), reached its peak at 18 months (due to inadequate health care and the feeding knowledge of mothers), and then the child effortlessly regained the lost weight [23].

The present study was conducted in 2016 and aimed to assess the knowledge and attitude of infants’ mothers from Bushehr (Iran) on food security using anthropometric indicators.

## Method

The present cross-sectional, descriptive-analytic study was conducted during 2016 in Bushehr, Iran. The study included mothers from Bushehr (Iran) having a child aged 1-2 years. The selection criterion was mothers who referred to the Comprehensive Health Service Centers in Bushehr for routine care and received early instructions related to complementary feeding. The sample size was estimated based on n = Z^2^_1-α/2_P(1-P)/d^2^ formula in accordance with previous studies in the field of knowledge and practice [24] and food security in Iran [25]. As part of the sampling process, all Comprehensive Health Service Centers (*n* = 10) in the city of Bushehr were visited. After counting the eligible mothers of each center, a sample size was randomly considered for each center. Following 3 months of sampling, the highest and lowest number of samples associated with each center was 64 and 21, respectively. The inclusion criteria were non-diagnostic neurological disorders and chronic musculoskeletal diseases of the mother, birth weight of the child > 2.5 kg and < 4 kg, starting complementary feeding after 6 months of age, a child with no acute and chronic gastrointestinal diseases, or other chronic diseases affecting feeding based on the physician opinion.

The data were collected using the demographic information form, knowledge questionnaire, attitude questionnaire, Radimer/Cornell questionnaire for food security, and anthropometric indicators of the child. A dedicated demographic information form comprising 23 questions, designed according to the demographic variables required in the research, was developed. The design and psychometric measurement of knowledge and attitude questionnaires on food security, according to food access and usage were done based on the Waltz method (2010) [26]. This was carried out in four stages:

1. Definition of food security and complementary feeding of children based on several literature reviews.
2. Design of the questionnaire items according to the three areas of food security (availability, access, and utilization) as well as national and international studies. The knowledge questionnaire had 20 items with 3-point Likert scale (right, wrong, I do not know) and the attitude questionnaire had 26 items with 5-point Likert scale (from totally agree to totally disagree).
3. Determination of the face and content validity of both questionnaires. The face validity of both knowledge and attitude questionnaires was measured by 10 mothers across all educational groups. Finally, the coefficient of face validity for all questions was higher than 1.5. The content validity of questionnaires was measured by 12 individuals (specialized in the fields of nutrition, social medicine, nursing, and public health education) using the Lawshe table. In content estimation, knowledge was scaled by CVR = 0.95 and CVI = 0.95, respectively. Moreover, attitude was scaled by CVI = 0.94 and CVR = 0.91, respectively.
4. The reliability of the questionnaire was measured by Cronbach’s alpha coefficient. The conditions used for the coefficient in a sample of 30 mothers for the knowledge and attitude sections were 0.7 and 0.76, respectively. It should be noted that the knowledge and attitude questionnaires had 2 and 4 negative items, respectively. The knowledge and attitude questionnaires were scored according to previous studies (KAP) [27] and FAO guideline for feeding knowledge, attitude, and performance [28]. Mothers who correctly answered 70% (14 out of 20 scores) and more of the knowledge questions were considered to have the desired knowledge. Mothers who answered < 70% of the questions correctly were considered to have inadequate or undesired knowledge (I do not know and false = 0, I know = 1). In the case of attitude questionnaire, mothers who answered 70% of the questions as agree or totally agree (18 out of 26 scores) were considered to have a positive or desired attitude. Mothers who received scores less than 18 were considered to have a negative and/or undesired attitude (I totally disagree and no idea = 0, I totally agree and agree = 1). Note that the scoring of the negative questions was inverted (I do not know and true = 0, wrong = 1).

The 16-item Radimer/Cornell questionnaire was used to measure food security [29]. Each item included three choices (not true, sometimes true, and most often true) and measured in three segments: household, individual, and infant (child) food security within the recent year. If the respondent marks all items as not true, the food security of household will be at the household level. A household with food insecurity should positively (sometimes true, and most often true) answer to one or more questions (items 1-8) and negatively (not true) answer to other items (9-16). The respondent should mark items 9-13 as not true in terms of food security at the individual level. Note that items 14-16 measure child’s food insecurity. Therefore, the answer “not true” means food security of the child whereas positive answer means food insecurity and hunger of the child. Internal consistency of the questionnaire was 0.89, 0.82, and 0.79 for food security of the household, individual, and child, respectively [30]. The reliability of the questionnaire was again measured and the Cronbach’s alpha was 0.92 in 30 qualified Iranian samples.

Anthropometric indicators of weight, height, and weight-for-height (WFH)were measured in accordance with the z-score benchmark of the World Health Organization. Weight-for-age index (WFA) was divided into three groups of underweight (− 3 ≤ z-score < − 2), normal (− 2 ≤ z-score ≤ + 1), and overweight (above + 1). The height-for-age (HFA) was divided into four groups of severe short height (less than − 3 z-score), short height (− 3 ≤ z-score < − 2), normal (− 2 ≤ z-score ≤ + 3), and tall (higher than + 3). The weight-for-height index (WFH) was divided into 5 groups of lean (less than − 2), normal (− 2 ≤ z-score ≤ + 1), overweight (+ 1 < z-score ≤ + 2), weight gain (+ 2 < z-score ≤ + 3), and obese (> + 3). The weight of the children was measured using a measuring scale with weighing precision of 5 g and control weights of 50, 100, and 1000 g. The measurement was carried out daily in sleeping or sitting position after having removed the excess clothes. The height was also measured in sleeping or standing height position using a fabric meter with a precision of 0.1 cm. The shoes, socks, and other accessories of the child were removed before the measurement. The body of the child was then held in position and the moving piece was attached to the child’s foot. If the child did not stretch, the height was measured in the standing position and 0.7 cm was added to determine the height [31].

### Ethical considerations

For the purpose of sampling, following the introduction of the research, the goals and duties of the participants during the study were clarified. The confidentiality of the information, including names and personal issues of the participants was stated. A written informed consent (e.g. subject to an active and optional presence, optional withdrawal in case of unwillingness to continue with the research, no liability due to damages caused by the research, and no reimbursement of personal expenses) was signed by the participants. The study was supported by the Research Council of Bushehr University of Medical Sciences and approved under the grant number 2015.5624 and the Research Ethics Committee (code: IR.BPUMS.REC.2016.9) of Bushehr University of Medical Sciences. The mothers who completed the questionnaires were awarded a complementary feeding manual containing food security issues, complementary feeding, and correct answers to the questions.

### Data analysis

The data were analyzed using the SPSS software, version 18.0. Descriptive statistics including average, mean, and standard deviation was used for demographic and anthropometric indicators. Spearman test was used to determine the relationship between knowledge, attitude, and food security questionnaires. Chi-square test and multinomial regression analysis were used to determine the relationship between anthropometric index and food security. Fisher’s exact number and logistic regression were used to determine the relationship between the anthropometric indicators with knowledge and attitude at a significant level of 0.05 and OR with 95% CI.

## Results

A total of 400 mothers entered the study. Their mean age was 29.53 ± 4.92 years, housewife (81%), employed (19%), high school diploma (41%), university degree (43.3%), married (98%), and 98% were not covered by any support organization. The mean age of the children was 16.44 ± 3.96 months and mean birth weight was 3.2 ± 0.43 kg (Table 1).

**Table 1:** Demographic indicators in a sample of 400 households with children aged 1-2 years

Variable			Frequency	%
Mother’s job*	Housewife		324	81
	Employed	Employee	73	18.3
		Housewifery	47	11.8
		Self-employed	1	3
Mother’s education	Illiterate		5	1.3
	Primary education		18	4.5
	Under diploma		40	10
	Diploma		164	41
	Bachelor and higher		173	43.3
Father’s education	Illiterate		2	0.5
	Primary education		29	7.3
	Under diploma		51	12.8
	Diploma		153	38.3
	Bachelor and higher		165	41.3
Father’s job	Employee		172	43
	Worker		33	8.3
	Self-employed		192	48
	Unemployed		2	0.5
Income	< 5 million IRR		49	12.2
	5-10 million IRR		133	33.3
	10-20 million IRR		160	40
	20 million IRR		58	14.5
Type of house	Owner		218	54.5
	Rented		151	37.8
	Living with other family members		30	7.5
Marital status	Married		395	98.8
	Divorced		1	0.3
	Widow		2	0.5
	Living apart		2	0.5
Under coverage of supportive organ	Yes		8	2
	No		392	98
Child sex	Girl		215	53.8
	Boy		185	46.3
Number of children	1		165	41.3
	2		173	43.3
	3		54	13.5
	4		6	1.5
	5		1	0.3
	6		1	0.3

In terms of knowledge, 74 (18.5%) mothers had undesired knowledge of food security in complementary feeding and 326 (81.5%) had the desired knowledge. Based on the results on attitude, 42 (10.5%) mothers had a negative attitude towards food security and 358 (89.5%) had a positive attitude. Based on the Radimer/Cornell questionnaire, the rate of food insecurity of the household, individual, and child levels was 51.5, 22.3, and 11.3%, respectively (results not shown in a table). Spearman correlation coefficient showed a significant positive relationship between knowledge and attitude (*r* = 0.26, *P* = 0.0001) and between knowledge and household food security (*r* = 0.11, *P* = 0.02) (Table 2).

**Table 2:** The relationship between food security and maternal knowledge and attitude in complementary feeding

Variable	Knowledge		Attitude		Individual food security		Child hunger	
	r	P	r	P	r	P	r	P
Attitude	0.26	**0.0001**	–	–	–	–	–	–
Individual food security	0.05	0.25	0.05	0.29	–	–	–	–
Child hunger	0.03	0.49	−0.19	0.70	0.66	**0.0001**	–	–
Household food insecurity	0.11	**0.02**	0.05	0.29	0.50	**0.0001**	0.34	**0.0001**

In relation to anthropometric indicators, the results showed that 71.5% of the children had normal weight, 26% were overweight to obese, and only 2.5% had weight loss. The mean weight of the children was 10.47 ± 1.66 kg (min: 7.50 kg, max: 18.91 kg). In measuring the height-for-age index, only three children had severe short height and 93.5% had a height within the normal range (80.16 ± 4.92). In terms of weight-for-height index, 72.75% had a weight for normal height and the remaining 27.25% (i.e. about a quarter of the children aged 1-2 years) fell under the risk category of overweight to obese (Table 3).

**Table 3:** Z-score for the height-for-age (HFA), weight-for-age (WFA), and weight-for-height (WFH) of the children aged 1-2 years (*n* = 400)

Anthropometric Indicators	Frequency	(%)
WFA		
Underweight	10	2.5
Normal	286	71.5
Overweight	104	26
HFA		
Sever stunting	3	0.8
Stunting	15	3.8
Normal	374	93.5
Tall	8	2
WFH		
Wasting	3	0.75
Normal	291	72.75
Risk of overweight	78	19.5
Overweight	17	4.25
Obese	11	2.75

The logistic regression between knowledge and weight-for-height index showed that the risk of obesity in children whose mothers did not have adequate knowledge was twice as high as those being lean and having a normal weight (OR = 2.33, *P* = 0.04). Also, the risk of short height in children whose mothers did not have adequate knowledge of food security was more than 4 times in comparison with the normal height (OR = 4.87, *P* = 0.001). The risk of obesity in children whose mothers had a negative attitude towards food security was more than 2 times in comparison with the normal weight (OR = 2.91, *P* = 0.03). No relationship was found between other anthropometric indicators to both knowledge and attitude (Table 4).

**Table 4:** Logistic regression analysis between mother’s insufficient knowledge and negative attitude with child (1-2 years) anthropometric indicators (n = 400)

Anthropometric indicators	Knowledge (insufficient)			Attitude (negative)		
	OR”	CI●	*P*-value	OR	CI	*P*-value
WFH^a^						
< + 2 Z-score^d^		1			1	
≥ + 2 Z-score^e^	2.33	1.01-5.41	0.04	2.91	1.09-7.71	0.03
HFA^b^						
< −2 Z-score^f^	4.87	1.86-12.75	0.001	0.56	0.15-2.05	0.38
≥ −2 Z-score^g^		1			1	
WFA^c^						
≤ + 1 Z-score^h^		1			1	
> + 1 Z-score^i^	0.81	0.45-1.48	0.51	1.49	0.75-2.95	0.25

^a^Weight-for-height

^b^Height-for-age

^c^Weight-for-age

^d^Wasted, normal, risk overweight

^e^Overweight and obese

^f^Stunting

^g^Normal and tall

^h^Underweight and normal

^i^Overweight

OR”: Odds ratio

CI●: Confidence interval (95%)

The multinomial regression between the food security and anthropometric index showed that the odds of weight gain in food secure households was 0.6 times higher than the food insecure households (OR = 0.61, *P* = 0.03). Also, the odds of weight gain in children without hunger (food secure) was 0.4 times higher than that of children with starvation (OR = 0.4, *P* = 0.04) (Table 5).

**Table 5:** Multinomial regression analysis between food security and anthropometric indicators of insecure to secure (*n* = 400)

Anthropometric indicators	Household food security			Individual food security			Child hunger		
	OR	CI	P	OR	CI	P	OR	CI	P
WFA^a^									
Normal	1			1			1		
Overweight	0.61	0.38-0.96	**0.03**	0.91	0.52-1.57	0.74	0.40	0.16-0.97	**0.04**
Underweight	1.25	0.34-4.52	0.73	0.85	0.17-4.10	0.84	0.72	0.89-5.88	0.76
WFH^b^									
Normal	1			1			1		
Risk of overweight	0.68	0.41-1.13	0.14	0.62	0.31-1.21	0.16	0.45	0.17-1.20	0.11
Overweight and obese	1.02	0.47-2.23	0.94	1.89	0.83-4.30	0.12	0.51	0.11-2.24	0.37

^a^Weight-for-age

^b^Weight-for-height

## Discussion

Based on the findings of the present study, the majority of the mothers had the desired knowledge and positive attitude towards food security in complementary feeding. This could be the result of preliminary instructions given at all Comprehensive Health Service Centers before and during complementary feeding. The knowledge of food security was purely related to the dimension of household food security of a family and not to the individual/child level of food security. As an interpretation, it can be argued that knowledge is as effective as controlling food insecurity that has not involved the child. Moreover, it seems that parents were prepared to prevent food insecurity of their children even by self-sacrifice (e.g. hunger). Resorting to food security knowledge, they attempted alternative foods and tried to resolve all other issues affecting the health of their children. However, it seems that the knowledge of a mother, as a positive factor, does not support child’s food security when the severity of household insecurity triggers a child’s hunger and food accessibility. Household support at this stage, either by managing food crises or resorting to food aid, could be an appropriate solution to improve the situation.

A study conducted in India by Jain et al. [32] reported that the knowledge of mothers regarding breastfeeding and complementary feeding was at an acceptable level of 83%. In addition, a study conducted in Boroujerd (Iran) reported that the knowledge of mothers regarding complementary feeding was 61% and satisfactory [24]. A study conducted in Ethiopia by Abeshu et al. [33] reported an appropriate maternal knowledge of food security in supplemental nutrition.

The results of the present study indicated a positive attitude towards food security in complementary feeding by the mothers. A study conducted in Indonesia by Suparmi et al. [34] reported 50% positive feeding attitude of the mothers. According to Behroozeh et al. [35], attitude is among the factors affecting food security that should be addressed. They argued that the attitude towards the effect of food on health and the notion of food enjoyment relate to food security. Vereecken et al. [36] also concluded that positive feeding attitude of a mother is one of the main principles of feeding adequacy of children. In addition, feeding habits, the cost of food, health, and religious beliefs have been an effective framework for people in Bushehr to store and consume food. Even among poor and less educated people, there was a tendency towards food diversification (red meat, chicken, fish, shrimps, and grain) as a source of protein. They consider food diversification as an important contributor to health and enforce it even at the cost of distaste by a family member. Such religious tendency is even applied during the holy month of Ramadan when they prepare special food for distribution to the public [37].

The present study showed a positive and direct relationship between knowledge and attitude of the mothers. It indicates that increased knowledge of a mother would, in turn, improve her attitude. Since attitude is the most important factor determining feeding behavior [28], the behavior of the mothers in Bushehr would improve by increasing their knowledge and aligning their attitude. However, this is inconsistent with the findings of Suparmi et al. [34] who indicated that there was no relationship between knowledge and attitude.

In relation to the weight-for-height index, the alarming prevalence of weight gain (from overweight to obese) was about 26.6% (i.e. a quarter of the sample population). Compared to other studies, Poh et al. [38] reported 21.6% overweight and obesity in Malaysia. Rahmani et al. (2014) reported that obesity among the Iranian individuals under 18 years of age was only at 6.1% [39]. Increased weight and obesity in the early years of life can increase the risk of developing metabolic and cardiovascular diseases during adulthood. It also adversely affects the self-confidence of a child. The cohort study by Ostovar et al. on elderly in Busheh (Iran) highlighted the high prevalence of the tendency for cardiovascular diseases and metabolic syndrome [40]. In the present study, inappropriate knowledge and negative attitude towards food security were associated with an increased risk of obesity. A study conducted in Turkey by Yabancı et al. [41] showed that the higher the knowledge of the mothers, the more normal the children index will be. Heslot [42] concluded that despite the fact that Iranians do not suffer from hunger, the community moves towards obesity due to the changes in feeding patterns. It seems that increased weight, in addition to being affected by the knowledge and attitude of the mothers, is likely to be the result of inappropriate conduct of the mothers despite their adequate knowledge (e.g. consuming non-nutrient supplements, snacks, high-calorie foods with the main meal, irregular use of supplements beside breast milk, artificial juice drinks, and reduced physical exercise and child mobility due to local climate). Further investigation regarding the above is recommended.

A study conducted in Damavand (Iran) by Salarkia et al. also stated that despite their adequate knowledge, mothers had inappropriate conduct regarding food security in complementary feeding [16]. In a study by Dhurandhar, obesity was considered as an indication of food insecurity [43]. Based on the results of the present study, another related index to knowledge was the weight-for-height for which the risk in short height in children of mothers with inadequate knowledge was high. Shorter height is an important indicator for examining the feeding status of a child. Note that unlike weight, height is not rapidly adjusted and it takes a longer time. In a study on 1257 primary school children in Hamadan (2012), the level of education and knowledge of the mothers was considered as one of the causes associated with reduced short height [44]. In a study conducted in Ghana by Saaka [45], a significant relationship was found between the height-for-age index and the knowledge of the mothers. Abuya et al. also considered the education of a mother as one of the most important indicators of the height-for-age index of a child [46]. Based on the relationship between inadequate knowledge and obesity and short height, it is concluded that mothers with poor knowledge use non-nutrient foods that are only high in calories. Consequently, while a child may not feel hungry or craving is suppressed, such non-nutrient foods do not meet the needs of a child in terms of nutrition. Contrary to our expectations, the high prevalence of overweight and obesity in infants suggests that the knowledge and attitude of the mothers towards issues related to malnutrition and underweight seem to have increased due to prior instructions. Studies conducted in the past 10 years in Iran indicated a decrease in the prevalence of malnutrition in Iranian children. The trend for underweight during 1995 to 2004 showed an improved nutritional status of children under 5 years of age in Iran [47]. However, it is unlikely that issues related to weight gain and obesity have been considered since some Iranian parents perceive slight overweight as desirable [48].

## Conclusion

The present research, among the few studies conducted in Iran, assessed the knowledge and attitude of mothers towards food security in complementary feeding. Localized knowledge and attitude questionnaires were used to measure food security and examine anthropometric indicators of children in complementary feeding. It was shown that the majority of the participants had the desired knowledge and positive attitude towards food security in complementary feeding. In the face of various levels of food insecurity (household, individual, and child), there is a need for alternative approaches. In food secure families, increased food security could be achieved by increasing the level of knowledge on nutrition. However, nutritional packages and family support (economic) will reduce food insecurity in food insecure families. This topic requires further investigation. Besides, various observations (e.g. the alarming prevalence of weight gain in infants, the relationship between inadequate knowledge and negative attitude towards food security combined with an increased risk of obesity, and the short stature of infants) indicated the need to determine other factors associated with food insecurity, including the conduct by the mothers in complementary feeding. In addition, due to the low number of lean infants, we combined the lean with normal-weight infants in order to estimate logistic regression. Therefore, a case-control study is recommended to address this limitation.

## Abbreviations

CVI: Content validity index
CVR: Content validity ratio
HFA: Height-for-age
WFA: Weight-for-age
WFH: Weight-for-height
